# Is Short Therapy an Appropriate Regimen for Children and Young Adolescents with Drug-Susceptible Tuberculosis?

**DOI:** 10.3390/ph19050721

**Published:** 2026-05-01

**Authors:** Susanna Esposito, Valentina Fainardi, Beatrice Rita Campana, Gaia Giorgia Arnesano, Nicola Principi

**Affiliations:** 1 Pediatric Clinic, Department of Medicine and Surgery, University Hospital of Parma, 43126 Parma, Italy; valentina.fainardi@unipr.it (V.F.); beatricerita.campana@unipr.it (B.R.C.); gaiagiorgia.arnesano@unipr.it (G.G.A.); 2 Università degli Studi di Milano, 20122 Milan, Italy; nicola.principi@unimi.it

**Keywords:** pediatric tuberculosis, short-course therapy, treatment duration, drug-susceptible tuberculosis, antitubercular therapy, treatment adherence

## Abstract

**Background:** Tuberculosis (TB) remains a major cause of morbidity and mortality among children worldwide, with approximately one million new pediatric cases annually. The conventional treatment for drug-susceptible TB has long relied on a 6-month multidrug regimen, which is highly effective but associated with challenges in adherence, toxicity, and healthcare burden. **Objectives:** To evaluate whether short-course therapy is an appropriate regimen for children and young adolescents with drug-susceptible TB, with particular focus on its efficacy, safety, and applicability in different clinical contexts. **Methods:** A structured narrative review of the literature was conducted, including randomized controlled trials, observational studies, and international guidelines addressing treatment duration in children and young adolescents with drug-susceptible TB. Evidence was synthesized focusing on children and young adolescents <16 years with drug-susceptible TB treated with short-course regimens compared to standard therapy. **Results:** A shorter treatment regimen, particularly 4-month courses, has been investigated as an alternative to standard therapy in the pediatric population with drug-susceptible TB. Children often present with paucibacillary and non-severe forms of TB, providing a biological rationale for treatment shortening. Evidence from a randomized controlled trial has demonstrated that a 4-month regimen is non-inferior to the standard 6-month therapy in children and young adolescents with non-severe, drug-susceptible TB. These findings have informed recent international guideline updates, which now recommend short therapy in carefully selected patients. However, a short regimen is not appropriate for infants younger than 3 months, children with severe or complicated TB, extrapulmonary disease such as central nervous system involvement, or those with drug-resistant TB. The overall quality of evidence remains moderate, and long-term relapse data are still emerging. **Conclusions:** Short-course therapy represents a promising but selective strategy in pediatric drug-susceptible TB management. It offers potential advantages, including improved adherence, reduced drug toxicity, and lower healthcare costs. However, its safe implementation requires accurate patient selection, access to appropriate diagnostic tools, and structured follow-up. Careful application within clearly defined clinical criteria is essential to ensure optimal outcomes.

## 1. Introduction

Tuberculosis (TB) continues to pose a substantial public health challenge worldwide, despite decades of control efforts [1]. While often perceived as a disease of adults, TB significantly affects children and young adolescents, particularly in high-burden regions [2,3]. Pediatric TB accounts for a considerable proportion of global cases and is associated with significant morbidity, long-term complications, and mortality, especially in young children and those with delayed diagnosis [3,4].

One of the major challenges in pediatric TB is its under-recognition. Children frequently present with non-specific clinical manifestations such as persistent cough, low-grade fever, weight loss, and fatigue [5,6,7]. These symptoms overlap with other common pediatric conditions, making early diagnosis difficult [7,8]. Furthermore, microbiological confirmation is often lacking due to the paucibacillary nature of the disease and difficulties in obtaining sputum samples [5,9]. As a result, many cases are diagnosed clinically, which may lead to both under- and over-treatment [6].

Historically, pediatric TB management has relied heavily on evidence derived from adult populations [10]. The standard 6-month treatment regimen has remained largely unchanged for decades, reflecting its proven efficacy in achieving cure and preventing relapse [11]. However, this “one-size-fits-all” approach does not fully account for the biological and clinical differences between adult and pediatric TB [9,10].

The prolonged duration of standard therapy presents several challenges. Adherence to treatment over six months can be difficult, particularly in children who depend on caregivers for medication administration [12,13]. Treatment fatigue, adverse drug effects, and social factors such as limited access to healthcare services can all contribute to incomplete therapy [12]. In turn, poor adherence increases the risk of treatment failure, relapse, and the development of drug resistance [13,14].

In recent years, there has been growing recognition that pediatric TB is a distinct disease entity [6,8]. Children often present with non-severe, localized forms of TB, characterized by lower bacterial burden and less extensive tissue damage [9,10]. These features suggest that shorter treatment durations may be sufficient in many cases, without compromising efficacy [15].

Advances in clinical research, including well-designed randomized trials, have begun to challenge the traditional paradigm of fixed 6-month therapy [15,16]. The concept of individualized treatment duration based on disease severity and patient characteristics is gaining traction [17]. Short-course therapy, typically lasting 4 months, has emerged as a potential alternative for selected pediatric patients [15].

However, the adoption of shorter regimens raises important questions [17,18]. Can efficacy be maintained with reduced treatment duration? What are the risks of relapse or acquired resistance? Which patients are suitable candidates? How can these regimens be implemented safely in diverse healthcare settings? How have countries and programmes successfully translated evidence on short-course regimens for non-severe pediatric TB into policy and routine practice? This manuscript aims to address these questions by critically evaluating the role of short therapy in children and young adolescents <16 years with drug-susceptible TB. It explores the biological rationale, reviews the available clinical evidence, examines current guideline recommendations, and discusses the practical and programmatic implications of treatment shortening.

## 2. Methods

This manuscript was developed as a narrative review with a structured approach to evidence synthesis, aimed at evaluating the appropriateness of short-course therapy for pediatric TB. The methodology was informed by principles commonly applied in systematic reviews and guideline appraisal, with a focus on clinically relevant questions regarding treatment duration in children.

The review defined the population as children and young adolescents aged 0 to 16 years with pulmonary or extrapulmonary tuberculosis. The intervention of interest was short-course anti-tubercular therapy, generally defined as treatment lasting four months or less, while the comparator consisted of standard treatment regimens of six months or longer. The primary outcomes considered included treatment success, relapse, mortality, adherence, adverse events, and the development of drug resistance. This framework allowed for a structured evaluation of the available evidence and facilitated comparison across studies and guideline recommendations.

A comprehensive literature search was conducted using major electronic databases, including PubMed/MEDLINE, Embase, and the Cochrane Library. In addition, relevant clinical guidelines and policy documents were identified through institutional websites such as those of the World Health Organization (WHO), the National Institute for Health and Care Excellence (NICE), the Centers for Disease Control and Prevention (CDC), and the Canadian Tuberculosis Standards.

The search strategy combined keywords and Medical Subject Headings (MeSH) related to pediatric TB and treatment duration, including terms such as “pediatric tuberculosis,” “childhood TB,” “short-course therapy,” “treatment duration,” “SHINE trial,” and “tuberculosis guidelines.” The search primarily focused on studies published from 2000 onwards, although earlier landmark studies were included when relevant to provide historical context.

Studies were included if they focused on pediatric populations or provided subgroup analyses relevant to children, evaluated treatment duration or short-course regimens, and reported clinically meaningful outcomes such as cure, relapse, or adverse events. Priority was given to randomized controlled trials, systematic reviews, and high-quality observational studies. Clinical guidelines were included if they demonstrated methodological rigor, as assessed through established tools such as AGREE II, and if they were based on evidence-based frameworks such as GRADE.

Studies that focused exclusively on adult populations without clear applicability to pediatric TB were excluded, unless they provided important comparative or mechanistic insights relevant to treatment duration.

Relevant data were extracted from the selected studies, including study design, population characteristics, details of the interventions and comparators, outcomes measured, and key findings. Particular emphasis was placed on randomized controlled trials evaluating short-course therapy, such as the SHINE trial, as well as on major international guideline recommendations.

A qualitative synthesis of the evidence was performed, integrating findings across studies and guidelines. The evidence was organized into thematic domains, including pathophysiological rationale, clinical trial evidence, guideline recommendations, advantages and limitations of short therapy, and considerations for special populations.

The quality of evidence was assessed based on study design, methodological rigor, and consistency of findings across studies. Given the limited number of pediatric-specific randomized trials, particular attention was paid to the consistency of findings and their applicability to clinical practice.

International guidelines, including those from the WHO, NICE, CDC, and Canadian authorities, were systematically reviewed to identify recommendations related to treatment duration in pediatric TB. Recommendations were categorized according to their applicability, including whether they could be directly adopted, adapted, or required contextual interpretation based on local resources and epidemiology.

As a narrative review, this study is subject to inherent limitations, including potential selection bias and the absence of formal quantitative meta-analysis. Although a structured and systematic approach was used, the synthesis relies partly on expert interpretation of the available evidence. Furthermore, heterogeneity in study populations, definitions of disease severity, and outcome measures may limit direct comparability across studies and reduce the generalizability of conclusions.

## 3. Pathophysiological Basis for Short Therapy in Children

The rationale for shortening TB treatment in children and young adolescents is rooted in fundamental differences in disease pathophysiology compared to adults [9,19]. Pediatric TB is typically a manifestation of primary infection rather than reactivation disease [9]. As a result, it tends to be less destructive and more localized [19].

One of the defining features of pediatric TB is its paucibacillary nature. Children generally harbor fewer *Mycobacterium tuberculosis* organisms, which reduces both disease severity and infectiousness [4,9]. This lower bacterial burden may also translate into a reduced need for prolonged antimicrobial therapy to achieve sterilization [20].

In contrast to adults, children rarely develop cavitary lung lesions, which are associated with high bacillary loads and persistent bacterial reservoirs [19]. Cavities create microenvironments where drug penetration may be suboptimal, necessitating longer treatment durations [20]. The relative absence of cavitation in children supports the feasibility of shorter regimens [9].

Another important consideration is the spectrum of disease presentation. Pediatric TB often involves intrathoracic lymph nodes, minimal pulmonary infiltrates, or limited pleural disease [9,18]. These forms are generally more responsive to treatment and less prone to relapse [17]. Moreover, children have a lower risk of transmitting TB, reducing the public health implications of incomplete treatment [21].

The host immune response also differs between children and adults. While younger children may have immature immune responses, they often exhibit less immunopathology, resulting in less tissue destruction [22]. However, this can be a double-edged sword, as severe disseminated disease may occur in infants and immunocompromised children [4].

Pharmacokinetics and pharmacodynamics of anti-TB drugs also vary in children, influencing drug exposure and treatment outcomes [23,24]. Advances in pediatric dosing have improved drug efficacy, further supporting the possibility of treatment shortening [23].

Despite these favorable factors, not all children and young adolescents are suitable for short therapy. Severe disease, high bacterial burden, or immunosuppression may necessitate longer treatment [11,25]. Therefore, the biological rationale supports short therapy only in carefully selected cases.

## 4. Evidence from Clinical Trials

The movement toward shorter treatment regimens for pediatric TB has been driven primarily by emerging evidence from randomized controlled trials (RCTs), supported by observational studies and extrapolation from adult data [17,18]. Historically, the evidence base for pediatric TB treatment has been limited, with most therapeutic strategies derived from adult studies conducted in the mid-20th century [20]. In recent years, however, dedicated pediatric trials have begun to address this gap, providing more robust data to inform clinical decision-making [15].

The study in this context is the SHINE trial (Shorter Treatment for Minimal Tuberculosis in Children), published in 2022 [15]. This multicenter, open-label, non-inferiority RCT was conducted across several high TB-burden countries, including Uganda, Zambia, South Africa, and India [15]. The trial enrolled children and young adolescents under 16 years of age with non-severe, drug-susceptible TB [15]. Non-severe disease was carefully defined to include children and young adolescents with smear-negative pulmonary TB, intrathoracic lymph node disease without significant airway obstruction, and limited pulmonary involvement without cavitation [9,15]. Table 1 shows the definition of non-severe vs. severe pediatric TB.

Participants were randomized to receive either the standard 6-month regimen (2 months of isoniazid [H], rifampicin [R], and pyrazinamide [Z] with or without ethambutol [E], followed by 4 months of H and R) or a shortened 4-month regimen (2 months of HRZ ± E followed by 2 months of HR) [15]. Table 2 summarizes standard vs. short therapy.

The primary endpoint was a composite outcome including treatment failure, relapse, death, or loss to follow-up within a defined period [15].

The SHINE trial demonstrated that the 4-month regimen was non-inferior to the standard 6-month regimen, with comparable rates of favorable outcomes in both groups [15]. Importantly, relapse rates were low and similar between arms, providing reassurance regarding the durability of treatment success [15]. Adverse events were also comparable, with no significant increase in toxicity observed in the shorter regimen [15]. These findings represent a landmark shift in pediatric TB management, providing the first high-quality evidence supporting treatment shortening in children [15].

However, several important considerations must be highlighted when interpreting the SHINE trial results. First, the study population was highly selected, including only children and young adolescents with well-defined non-severe disease [15]. As such, the findings cannot be generalized to children with more severe or complicated forms of TB [9]. Second, the trial was conducted in controlled research settings with close monitoring and high levels of adherence, which may not fully reflect real-world conditions [18]. Third, while follow-up extended beyond treatment completion, longer-term outcomes beyond two years remain less well characterized [17].

In addition to the SHINE trial, other studies have explored the potential for treatment shortening, particularly through the use of novel drug combinations. The trial by Dorman et al. (2021), although primarily conducted in adults and adolescents, evaluated a 4-month regimen incorporating rifapentine and moxifloxacin [16]. This study demonstrated that rifapentine- and moxifloxacin-containing regimens could achieve non-inferior outcomes compared to standard therapy in drug-susceptible TB [16]. While pediatric data from this study are limited, the results have influenced the broader discussion on treatment shortening and the role of newer agents [16].

Further evidence comes from earlier adult trials investigating fluoroquinolone-based regimens, such as REMoxTB, RIFAQUIN, and OFLOTUB [26,27,28]. These studies generally failed to demonstrate non-inferiority of 4-month regimens in unselected adult populations, largely due to higher relapse rates [26,27,28]. However, these trials included patients with more severe disease, including cavitary TB, highlighting the importance of disease severity in determining appropriate treatment duration [26,27]. The contrast between adult and pediatric findings underscores the unique characteristics of children and young adolescents with drug-susceptible TB and supports a more tailored approach [9,15].

Observational studies and systematic reviews have also contributed to the evidence base, although their findings are more heterogeneous [17,18]. Many of these studies suggest that shorter regimens may be effective in selected populations but are limited by methodological constraints, including small sample sizes, lack of control groups, and variability in outcome definitions [17].

Despite these advances, the overall quality of evidence supporting short therapy in children remains limited. This reflects not only the limited number of pediatric RCTs but also the challenges inherent in conducting such studies, including ethical considerations, diagnostic uncertainty, and variability in disease presentation [10].

Another important limitation is the relative lack of data in specific subgroups. Infants under 3 months of age and those with extrapulmonary or disseminated TB are underrepresented in clinical trials [29,30]. Similarly, data on drug-resistant TB in children and young adolescents are sparse, and treatment recommendations in these populations rely heavily on adult studies and expert opinion [13,31]. Table 3 shows the eligibility criteria for short therapy of pediatric TB.

Adherence and programmatic factors also influence the interpretation of clinical trial data. In research settings, adherence is typically closely monitored, and patients receive comprehensive support [18]. In routine clinical practice, adherence may be lower, potentially affecting treatment outcomes [12]. Therefore, the effectiveness of short therapy in real-world settings may differ from trial results [14].

Finally, the issue of relapse remains a central concern. While short-term outcomes appear comparable between short and standard regimens, long-term follow-up is essential to fully assess relapse risk [17,18]. Current data suggest that relapse rates are low in selected populations, but continued surveillance is necessary to confirm these findings over time [18].

In summary, the evidence from a clinical trial supports the use of short-course therapy in carefully selected children and young adolescents with non-severe, drug-susceptible TB [15]. The SHINE trial provides the strongest evidence to date, demonstrating non-inferiority of a 4-month regimen in this population [15]. However, the applicability of these findings is limited by strict eligibility criteria, and significant gaps remain in the evidence base. Further research is needed to expand the applicability of short therapy, improve patient selection, and ensure long-term safety and efficacy [17].

## 5. Guideline Recommendations

The incorporation of short-course therapy into pediatric TB management has been driven by evolving evidence and reflected in recent updates of international and national guidelines [11,29,30,31,32,33]. Organizations such as the WHO, along with regional bodies and national agencies, have begun to recommend shorter regimens for carefully selected pediatric populations [29,30].

Current guidelines suggest that a 4-month treatment regimen may be considered for children and young adolescents aged 3 months to 16 years with non-severe, drug-susceptible TB [29,30]. The recommended regimen typically consists of an intensive phase of 2 months with H, R, and Z, with or without E (2HRZ(E)), followed by a continuation phase of 2 months with H and R (2HR) [29]. This shortened regimen is based primarily on evidence from the SHINE trial and subsequent evaluations [15,17].

A critical component of guideline implementation is the precise definition of “non-severe” TB. According to current recommendations, non-severe disease includes [29,30]: intrathoracic lymph node TB without airway obstruction; non-cavitary pulmonary TB confined to a limited area (e.g., a single lobe); paucibacillary disease without extensive radiological involvement; uncomplicated pleural effusion.

Conversely, children and young adolescents with any of the following features are excluded from short therapy [29,30]: cavitary pulmonary TB; extensive lung involvement or multilobar disease; miliary or disseminated TB; persistent bacteriological positivity after the intensive phase; prior TB treatment within the preceding two years.

These criteria highlight the importance of accurate disease classification, which requires access to appropriate diagnostic tools, including chest radiography and, when available, microbiological testing [5,8,34].

Age is another important consideration. Infants younger than 3 months are not eligible for short therapy due to the absence of evidence and the higher risk of severe disease and adverse drug effects [29,30]. In this population, the standard 6-month regimen remains strongly recommended [29]. Additionally, dosing adjustments may be necessary in very young infants to account for pharmacokinetic differences and potential toxicity [23].

Guidelines also address the role of short therapy in adolescents. In selected adolescents with drug-susceptible TB, particularly those over 12 years of age, alternative 4-month regimens incorporating rifapentine and moxifloxacin have been proposed [11,16]. However, these regimens are not universally available and may be limited by regulatory and logistical constraints (e.g., lack of drug availability in certain countries) [11].

For children and young adolescents with severe or complicated TB, the standard 6-month regimen (2HRZE/4HR) remains the cornerstone of treatment [11]. In some cases, particularly in cavitary disease or when microbiological response is delayed, extension of therapy to 9 months may be considered [11]. These recommendations are generally supported by stronger clinical experience, although the evidence base still includes a substantial proportion of expert opinion [11].

Extrapulmonary TB presents additional complexity. While some forms of extrapulmonary TB (e.g., uncomplicated lymph node disease) may be treated with standard durations, others require prolonged therapy [29,30]. For example: tuberculous meningitis often requires 6–12 months of treatment [29,30]; osteoarticular TB typically requires 12 months [29,30]; short-course therapy is not recommended in these settings due to the risk of severe complications and relapse [29,30].

Drug resistance is another critical factor influencing guideline recommendations. In children and young adolescents with isoniazid-resistant TB, treatment regimens must be modified to include additional agents such as fluoroquinolones, typically for a duration of at least 6 months [11]. For multidrug-resistant TB (MDR-TB), newer shorter regimens of approximately 6 months have been proposed, incorporating drugs such as bedaquiline, delamanid, and linezolid [35].

An important aspect emphasized in guidelines is the need for structured follow-up after completion of therapy. This is particularly relevant for children and young adolescents treated with short regimens, as early detection of relapse is essential [29,30]. Recommendations include close clinical monitoring in the first months after treatment completion, chest radiography and bacteriological testing if available, followed by continued follow-up for up to 12–24 months depending on disease severity and risk factors [30].

Implementation of guideline recommendations presents several challenges. In high-resource settings, access to diagnostic tools and specialist expertise facilitates accurate patient selection [11]. In contrast, in low-resource settings—where the burden of pediatric TB is highest—limited access to imaging, microbiology, and trained personnel may hinder appropriate application of short therapy [29]. This raises concerns about potential misclassification of disease severity and inappropriate use of shortened regimens [8]. Implementation considerations are critical for the safe scale-up of short-course therapy in children and young adolescents. In many settings, the diagnostic tools and specialist expertise needed to distinguish non-severe from severe pediatric TB are concentrated at secondary or tertiary level, whereas treatment delivery and longitudinal follow-up can often be provided effectively at primary health care (PHC) level [29,30]. A practical service-delivery model is therefore one in which children and young adolescents with suspected severe disease, diagnostic uncertainty, extrapulmonary involvement, very young age, drug resistance, comorbidities, or poor clinical response are assessed at a higher-level facility, while children and young adolescents classified as having non-severe, drug-susceptible TB can continue treatment and follow-up at PHC through clear referral and back-referral pathways [29,30]. To support this approach, standardized classification tools, simple job aids, and decision algorithms adapted for lower-level facilities are needed, ideally combined with remote clinical support, image review, or case discussion with district hospitals [29,30]. Hub-and-spoke or mentorship models, in which district or referral hospitals provide supportive supervision, training, and periodic case review for PHC teams, may help maintain diagnostic quality and confidence in decentralized care. Shared documentation systems—including referral forms, treatment cards, and, where available, electronic TB registers—are also essential to reduce loss to follow-up and ensure continuity across levels of care [29,30]. Finally, implementation should include quality-assurance mechanisms such as routine audit and feedback, review of referral appropriateness, treatment outcome monitoring, and accountability for post-treatment follow-up, so that programme managers can verify that decentralization improves access without compromising safety or outcomes [29,30]. Table 4 summarizes practical implementation considerations across levels of care.

Furthermore, variability in drug availability and regulatory approval can affect the feasibility of certain regimens [11,16].

In summary, current guideline recommendations support the use of short-course therapy in a carefully defined subset of children and young adolescents with non-severe, drug-susceptible TB [29,30]. These recommendations represent a significant shift toward more individualized treatment approaches but are accompanied by important caveats. The conditional nature of the recommendations underscores the need for careful clinical judgment, appropriate diagnostic support, and robust follow-up systems [11]. As additional evidence emerges, guidelines will likely continue to evolve, potentially expanding the role of short therapy in pediatric TB management [17].

## 6. Advantages of Short Therapy

Short-course therapy for pediatric drug-susceptible TB represents not only a reduction in treatment duration, but also a broader shift toward more patient-centered, efficient, and sustainable care [15,17]. Its potential advantages extend beyond clinical efficacy alone and involve multiple domains, including treatment adherence, drug safety, psychosocial well-being, healthcare system efficiency, and public health impact [17,18].

One of the most important potential benefits is improved adherence and treatment completion. Adherence to anti-tubercular therapy is especially challenging in children and young adolescents because treatment administration depends largely on caregivers [12,14]. Maintaining a 6-month regimen may be difficult because of treatment fatigue, competing family responsibilities, and socioeconomic barriers [12,36,37]. By reducing therapy to 4 months, the cumulative burden on both patients and caregivers may be lowered, with fewer doses, shorter follow-up, and earlier treatment completion [16]. This can improve motivation and reduce the risk of missed doses or premature discontinuation, which is particularly important because better adherence is associated with higher cure rates and lower risks of relapse and acquired drug resistance [14,17]. In this sense, shorter regimens are also more aligned with patient-centered care, as they take into account the practical realities faced by families [17].

A second major advantage is the reduction in cumulative drug exposure and, potentially, in treatment-related toxicity. Although first-line anti-TB drugs are usually well tolerated in children and young adolescents, they are still associated with adverse effects. Hepatotoxicity is a concern with isoniazid and pyrazinamide, rifampicin may cause gastrointestinal symptoms, hypersensitivity reactions, and drug interactions, and ethambutol carries a theoretical risk of optic neuritis [11]. A shorter regimen may decrease both the incidence and severity of adverse events by reducing the overall duration of exposure [24,26]. This is particularly relevant in young children, whose pharmacokinetics and drug metabolism differ from those of adults [23]. Fewer adverse effects may also indirectly improve adherence, since children and young adolescents are more likely to complete treatment and caregivers may feel more confident continuing therapy when toxicity is limited [12].

Short therapy may also offer important psychosocial and developmental benefits. Prolonged TB treatment can significantly disrupt a child’s and young adolescent’s normal life, affecting school attendance, social interactions, and family routines [4]. Repeated clinic visits, strict medication schedules, and monitoring requirements can place a considerable burden on both patients and caregivers [12]. A shorter regimen may allow children and young adolescents to return earlier to normal activities, reducing the psychosocial consequences of illness and supporting cognitive, emotional, and social development [4,15]. For caregivers, the shorter duration may reduce stress, time commitment, and even income loss related to repeated healthcare visits [12]. These benefits are especially relevant in vulnerable populations, where prolonged treatment can aggravate existing economic and social difficulties [3].

From a health-system perspective, shorter regimens may also provide significant economic and organizational advantages [1]. Direct costs may be reduced through lower medication use, fewer clinic visits, and less intensive monitoring [1]. Indirect costs, including transportation, caregiver time, and lost productivity, may also decrease [12]. In high-burden or resource-limited settings, shorter treatment may improve the efficiency of healthcare delivery by allowing providers to allocate limited resources across a larger number of patients [1]. In addition, simplified regimens may be easier to implement, monitor, and standardize within TB programs, thereby improving scalability and integration into existing health services [29].

At the public health level, shorter regimens may contribute to improved TB control. Better adherence and higher treatment completion rates reduce the likelihood of relapse and the development of drug resistance, both of which are major threats to TB management [13,14]. Although children are generally less infectious than adults, reducing the duration of disease and treatment may still have indirect benefits for transmission within households and communities [21]. Improved outcomes in pediatric TB may also reduce the long-term burden of TB-related morbidity [3]. In addition, shorter therapy may help reduce the stigma associated with TB, since a shorter treatment period may lessen the visibility and social impact of the disease on affected families [4].

Another important aspect is that short-course therapy fits with broader trends toward individualized and precision-based medicine [17]. Instead of applying the same treatment duration to all patients, clinicians can adapt therapy according to disease severity, patient characteristics, and risk factors [25]. This reflects recognition of the heterogeneity of pediatric TB and supports a more flexible and tailored approach to care [17,25].

Shorter regimens may also improve programmatic feasibility, particularly in decentralized or low-resource settings [29]. Simplified protocols can reduce logistical burdens related to drug supply, storage, and distribution, and they may facilitate community-based models of care delivered closer to the patient’s home [14,29]. At the same time, these benefits depend on proper patient selection and strict adherence to guideline criteria. If short therapy is used inappropriately, its advantages could be lost and adverse outcomes could occur [11,29].

Finally, short-course therapy may reduce caregiver burden, which is a crucial but sometimes underappreciated aspect of pediatric TB management. Caregivers are central to medication administration, clinic attendance, and monitoring for side effects [12]. A shorter regimen can reduce this responsibility more quickly, which may improve family well-being and indirectly support better adherence [12,14].

Overall, short-course therapy offers a wide range of potential advantages that go far beyond shortening treatment duration alone [15]. It may improve adherence, reduce toxicity, lessen psychosocial disruption, lower costs, simplify TB programs, and support broader public health goals [17,18]. However, these benefits can only be realized when short therapy is applied to appropriately selected patients and accompanied by careful monitoring and follow-up, so that treatment shortening does not compromise long-term outcomes (Table 5).

## 7. Limitations and Challenges

Despite encouraging evidence, short-course therapy for pediatric drug-susceptible TB has important limitations that must be considered before widespread implementation [15,17]. The main challenges are clinical, diagnostic, epidemiological, and programmatic, and they highlight the difficulty of translating trial evidence into routine practice [11,18]. Although short therapy has clear advantages, inappropriate use could compromise outcomes and weaken TB control efforts [13].

A major limitation is its restricted eligibility. Current recommendations apply only to children and young adolescents with clearly defined non-severe, drug-susceptible TB [29,30]. In practice, however, distinguishing non-severe from severe disease is not always easy, particularly in routine or resource-limited settings [25]. Severity classification often depends on clinical, radiological, and microbiological criteria, but access to imaging, microbiological confirmation, and specialist interpretation may be limited [5,8,34]. This creates a risk of misclassification, with potentially serious consequences if children and young adolescents with more extensive disease are treated with an insufficiently short regimen [17,18].

Another important issue is the limited generalizability of trial data. Studies such as SHINE provide high-quality evidence, but they were conducted in selected populations and under well-supported conditions, including close monitoring, high adherence, and standardized diagnostics [15,18]. These conditions may not reflect routine care, especially in low-resource settings where infrastructure, follow-up capacity, and access to services are more limited [1,11,29]. In addition, epidemiological differences across settings, including the burden of HIV or drug resistance, may affect the applicability of trial findings [3,15].

The evidence base is also limited in several important pediatric subgroups [10]. Infants younger than 3 months, children with malnutrition, extrapulmonary or disseminated TB, and some adolescent groups remain underrepresented in clinical trials [19,29,30]. Because these populations are often more vulnerable and at higher risk of severe disease or poor outcomes, recommendations for short therapy cannot be easily extended to them [4,29].

Relapse and long-term safety remain key concerns [17,18]. Although 4-month regimens have shown non-inferior short- and medium-term outcomes in selected patients, long-term data are still limited [15]. Even small increases in relapse could have important implications, particularly in high-burden settings, and recurrent disease may contribute to transmission and drug resistance [1,13,21]. For this reason, careful monitoring after treatment completion is essential [36].

Diagnostic limitations further complicate implementation. Pediatric TB is inherently difficult to diagnose, and microbiological confirmation is often not possible [5,6]. In many settings, access to chest radiography, Xpert MTB/RIF, culture, and expert interpretation remains limited [8,34]. Without reliable diagnostic support, both underdiagnosis and misclassification become more likely, making the use of short therapy less safe [6,11].

Programmatic and health-system constraints are equally important [29]. Introducing short therapy requires updated guidelines, staff training, reliable drug supply, and systems to ensure correct patient selection and continuity of care [1,11,29]. Long-term follow-up is especially challenging in settings with weak health information systems, limited resources, or high patient mobility [1,36]. Although shorter regimens may improve adherence, this is not guaranteed in real-world practice, where adherence is influenced by caregiver education, socioeconomic barriers, cultural factors, and access to care [12,14]. If adherence is poor, treatment failure or relapse may still occur [17].

Finally, drug resistance and ethical concerns must be taken into account. Short therapy is not appropriate for children and young adolescents with isoniazid-resistant or multidrug-resistant TB, who require modified and often longer regimens [11,35]. Undetected resistance may make shortened treatment ineffective and could contribute to further resistance [13]. Ethically, reducing treatment burden is valuable, but only if patient safety is preserved. This requires clear guidelines, adequate clinician training, and ongoing evaluation of outcomes to avoid overextending short therapy beyond the populations for whom it is supported [8,11,29].

Overall, short-course therapy is promising but must be implemented cautiously [15,17]. Its benefits can only be realized through accurate diagnosis, careful patient selection, strong follow-up systems, and continued research, so that treatment shortening improves care without compromising safety or long-term outcomes [1,11,29].

## 8. Special Populations

Although short-course therapy is a promising option for children and young adolescents with non-severe, drug-susceptible TB, its use is limited in several important pediatric subgroups [15,29,30]. These include patients with severe or complicated disease, extrapulmonary TB, drug-resistant TB, very young infants, immunocompromised children, malnourished children, and some adolescents [29,30]. In these groups, treatment often needs to be longer, more individualized, and more closely monitored [11].

Short therapy is generally not appropriate for children and young adolescents with severe or complicated TB, such as cavitary disease, extensive pulmonary involvement, miliary TB, or persistent bacteriological positivity after the intensive phase [11,25,29]. In these cases, the bacillary burden is higher and the risk of treatment failure or relapse is greater if therapy is shortened [8,17,20]. Current recommendations therefore favor the standard 6-month regimen, with extension to 9 months or longer when response is delayed [11].

Extrapulmonary TB also requires caution because the appropriateness of short therapy depends on the site and severity of disease [29,30]. While uncomplicated lymph node or limited pleural TB can usually be treated with standard 6-month regimens, short therapy has not been adequately studied in these forms and is not routinely recommended [29]. More severe forms, especially central nervous system TB and osteoarticular TB, require prolonged treatment because inadequate therapy may lead to serious sequelae such as neurological damage, deformity, or death [4,29,30].

Drug-resistant TB represents another major limitation [13,38,39,40,41]. Isoniazid-resistant and multidrug-resistant TB require alternative regimens that are usually longer, more toxic, and less well studied in children [11,13]. Although newer shorter regimens for MDR-TB are being explored, pediatric evidence remains limited, and management should be individualized and ideally undertaken in specialized centers [11,35].

Very young infants, especially those younger than 3 months, are another vulnerable group in whom short therapy is not supported by current evidence [29,30,42]. Because they are at higher risk of severe and disseminated disease and have distinct pharmacokinetic characteristics, standard 6-month therapy with careful dose adjustment remains recommended [23,29,42]. Similarly, children and young adolescents with immunocompromising conditions are more likely to develop severe or atypical TB and may have more complex management because of drug interactions and higher risks of treatment failure or relapse [4,11,29,30,43].

Other vulnerable groups also require caution. Malnourished patients may have impaired immunity, altered drug handling, and more severe disease, but evidence on short therapy in this population is still limited [3,23,29]. Adolescents represent a transitional group because their TB may resemble adult disease, with more cavitary lesions and higher bacillary loads, while psychosocial factors, stigma, and autonomy can complicate adherence [12,15,19,21]. Some may be eligible for short therapy, but careful assessment is necessary [11].

Overall, these special populations highlight that short-course therapy cannot be applied uniformly across all children and young adolescents with TB [25,29,30]. Treatment decisions must take into account disease severity, anatomical site, age, immune status, nutritional status, and the risk of drug resistance [11]. In many cases, specialist input is advisable. Expanding funding for global pediatric health and strengthening integrated TB services at the primary care level are also essential to ensure safe, effective, and equitable care for patients who are not candidates for simplified regimens [1].

## 9. Adherence and Follow-Up

Adherence to therapy and structured post-treatment follow-up are essential components of successful pediatric TB management [11,12]. Although shorter regimens may reduce treatment burden and improve completion rates, these benefits are not automatic and depend on clinical, social, and health system factors [14,17]. In fact, the use of short-course therapy makes careful follow-up even more important in order to confirm sustained cure and detect relapse early [29,30].

Adherence is particularly challenging in children and young adolescents because treatment depends heavily on caregivers for medication administration, clinic attendance, and follow-up [12]. With standard 6-month regimens, adherence often declines over time because of treatment fatigue, adverse effects, caregiver burden, and socioeconomic barriers [11,12]. Shorter regimens may be easier for families to complete and may therefore improve adherence [15]. However, adherence remains influenced by many factors, including the patient’s age and clinical condition, caregiver understanding and resources, and the accessibility and quality of healthcare services [1,12,14].

Improving adherence requires a multifaceted approach [12]. Key strategies include caregiver education, simplified regimens and child-friendly formulations, psychosocial support, decentralized models of care, and practical adherence support through community- or family-based approaches [1,4,14,23]. Digital tools such as SMS reminders, mobile applications, and electronic medication monitors may also be useful, especially for older children and adolescents [14]. Even with short therapy, families must understand that completing the full prescribed course remains essential, since missed doses may have a proportionally greater impact when treatment duration is reduced [11,17].

Follow-up after treatment completion is equally important, particularly for children and young adolescents treated with shortened regimens [29,30]. Current recommendations support structured monitoring in the early post-treatment period and continued follow-up for up to 12–24 months depending on the clinical context [29,30,34]. The aims of follow-up include detecting relapse or treatment failure, identifying residual disease or complications, monitoring growth and development, and assessing long-term sequelae such as impaired lung function [11,36]. Depending on the patient, follow-up may include clinical review, radiological reassessment, selected microbiological testing, and multidisciplinary evaluation in children with extrapulmonary disease [5,8,29,30,34].

Despite its importance, follow-up can be difficult to implement, especially in resource-limited settings [1]. Common barriers include patient mobility, socioeconomic difficulties, limited staff and infrastructure, weak information systems, and poor caregiver awareness of the need for ongoing monitoring [1,12]. These challenges underline the need to balance efficiency with safety: short therapy should not simply reduce treatment length, but should be accompanied by strengthened systems for adherence support and post-treatment surveillance [11,17,29].

Overall, adherence and follow-up should be integrated into the treatment plan from the beginning [11,12]. Short-course therapy may improve convenience and reduce burden, but its success depends on maintaining high adherence during treatment and ensuring robust follow-up afterward [14,17]. These elements are essential to preserve treatment efficacy, prevent relapse, and protect long-term outcomes in children and young adolescents with TB [11].

## 10. Future Directions

The evolving landscape of pediatric TB treatment highlights several critical areas for future research and clinical development [15,17]. While the introduction of short-course therapy represents an important step toward more patient-centered care, significant gaps remain that must be addressed before such regimens can be widely and confidently implemented across diverse populations and healthcare settings.

One of the foremost priorities is the expansion of high-quality pediatric-specific evidence. Despite recent advances, children and young adolescents remain underrepresented in clinical trials, and much of the current guidance is derived from limited datasets or extrapolated from adult populations [10,17]. Future studies should focus on enrolling larger and more diverse pediatric cohorts, including infants, adolescents, and children with comorbidities such as immunocompromising conditions or malnutrition. Particular attention should be given to populations in high-burden settings, where the epidemiology of TB may differ significantly and where healthcare infrastructure may influence treatment outcomes.

Another key research area is the refinement of disease classification. The current distinction between “non-severe” and “severe” TB is essential for determining eligibility for short therapy but remains somewhat imprecise in clinical practice [25]. Developing standardized, reproducible, and accessible criteria—potentially incorporating imaging, microbiological, and biomarker-based tools—would improve patient selection and reduce the risk of misclassification. In this context, advances in radiological techniques, molecular diagnostics, and host-response biomarkers hold significant promise [8,34].

The identification of reliable biomarkers to guide treatment duration represents a particularly important frontier [22]. Biomarkers that can accurately reflect bacterial burden, treatment response, and risk of relapse would enable truly individualized therapy [17]. Such tools could allow clinicians to tailor treatment duration to the specific needs of each patient, rather than relying on fixed regimens. This precision medicine approach has the potential to optimize outcomes while minimizing unnecessary drug exposure [23].

Pharmacological innovation also plays a central role in future developments [23,24]. The optimization of pediatric drug formulations, including dispersible and palatable preparations, is essential to improve adherence and ensure accurate dosing [23]. Furthermore, ongoing research into novel anti-TB agents and drug combinations may facilitate even shorter and more effective regimens [16,35]. The integration of newer drugs, such as rifapentine and bedaquiline, into pediatric protocols requires careful evaluation through dedicated clinical trials.

Another promising avenue is the exploration of ultra-short regimens, potentially lasting less than four months [17]. SMILE-TB (NCT06253715) is a phase 3, open-label, randomized, controlled non-inferiority trial in children with presumed drug-susceptible TB started in January 2025, with estimated completion in September 2027 and target enrollment of 860 participants. The trial is testing whether a 2-month regimen with isoniazid, rifapentine, pyrazinamide and moxifloxacin can be as safe and effective as the current 4–6-month standard of care in children under 10 years with TB in the lungs and/or lymph nodes, with or without HIV. While still experimental, these approaches could further reduce treatment burden and improve adherence [12]. However, rigorous evaluation of efficacy, safety, and long-term outcomes is necessary before such regimens can be recommended.

Implementation research is equally important. Even the most effective treatment regimens will have limited impact if they cannot be successfully integrated into real-world healthcare systems [1]. Studies should assess the feasibility, acceptability, and cost-effectiveness of short therapy in different settings, including low-resource environments. This includes evaluating healthcare worker training, supply chain management, and patient support systems.

Digital health technologies may also play a role in enhancing treatment adherence and monitoring [14]. Tools such as electronic medication monitors, mobile health applications, and telemedicine platforms could support caregivers and healthcare providers in managing pediatric TB more effectively.

Finally, long-term outcome data are needed to fully understand the implications of short therapy [17,18]. This includes not only relapse rates but also the impact on lung function, growth, neurodevelopment (particularly in cases of CNS involvement), and overall quality of life. Establishing robust surveillance systems and longitudinal cohorts will be essential to capture these outcomes.

In summary, while short therapy represents a promising advancement, its future development depends on a multidisciplinary effort encompassing clinical research, diagnostics, pharmacology, and health systems strengthening. Only through such comprehensive approaches can the full potential of treatment shortening in pediatric TB be realized.

## 11. Conclusions

The question of whether short therapy is an appropriate regimen for pediatric drug-susceptible TB reflects a broader shift toward more individualized and evidence-based approaches in infectious disease management [11,17]. The traditional 6-month treatment regimen has served as a reliable and effective standard for decades, providing high cure rates and forming the backbone of global TB control strategies [11]. However, it is increasingly clear that this uniform approach may not be optimal for all pediatric patients [9,25].

Emerging evidence, particularly from well-conducted randomized controlled trials, demonstrates that a 4-month treatment regimen can achieve outcomes comparable to standard therapy in children and young adolescents with non-severe, drug-susceptible TB [15,16]. These findings are supported by a strong biological rationale, including the typically lower bacillary burden and less extensive disease seen in pediatric populations [9,19]. As a result, short-course therapy has gained recognition as a viable option within carefully defined clinical parameters [29,30].

Nevertheless, the applicability of short therapy is inherently limited. It is not appropriate for all children and young adolescents, and its use must be restricted to those who meet strict eligibility criteria [29,30]. Children and young adolescents with severe or complicated TB, extrapulmonary disease involving critical sites such as the central nervous system, very young infants, and those with suspected or confirmed drug resistance require standard or extended treatment regimens [11,29,35]. In these cases, the risks associated with undertreatment outweigh the potential benefits of shorter therapy [17].

Moreover, the current evidence base, while encouraging, remains incomplete. The quality of evidence is classified as moderate, and important questions persist regarding long-term outcomes, relapse risk, and applicability across diverse populations. These uncertainties underscore the need for cautious implementation and continued research.

From a clinical perspective, the successful use of short therapy depends on several key factors. Accurate diagnosis and disease classification are essential to identify eligible patients [5,8,34]. Access to appropriate diagnostic tools, including radiological and microbiological methods, is therefore critical. In addition, strong systems for adherence support and follow-up must be in place to ensure treatment completion and early detection of relapse [12,36].

From a public health standpoint, short therapy offers significant potential benefits. Improved adherence, reduced treatment costs, and decreased healthcare burden could enhance the effectiveness of TB control programs [1]. However, these advantages can only be realized if short regimens are implemented thoughtfully and supported by robust health systems.

Ultimately, short therapy should be viewed not as a replacement for standard treatment but as a complementary option within a broader, patient-centered framework [17]. Its role is to provide a tailored approach for children and young adolescents with non-severe disease, balancing efficacy with feasibility and quality of life [25].

As the field continues to evolve, the integration of new evidence, technologies, and therapeutic strategies will further refine pediatric TB management. The goal is to move toward more precise, effective, and humane treatment approaches that address the unique needs of the pediatric population.

Overall, short therapy is an appropriate regimen for pediatric drug-susceptible TB—but only when applied judiciously, within clearly defined clinical criteria, and supported by careful monitoring and ongoing research.

## Figures and Tables

**Table 1:** Definition of Non-Severe vs. Severe Pediatric Tuberculosis (TB) in the SHINE Trial.

Feature	Non-Severe TB	Severe/Complicated TB
Pulmonary involvement	Limited (single lobe, non-cavitary)	Extensive, multilobar, or cavitary
Bacterial load	Paucibacillary	High bacillary burden
Radiology	Minimal infiltrates, lymph nodes	Cavitation, miliary, widespread
Clinical status	Mild symptoms	Severe symptoms
Microbiology	Smear-negative	Smear-positive

**Table 2:** Standard vs. Short Therapy for Pediatric Tuberculosis in the SHINE trial.

Characteristic	Standard (6 mo)	Short (4 mo)
Regimen	2HRZ(E)/4HR	2HRZ(E)/2HR
Adherence	Moderate	Improved
Relapse risk	Low	Comparable
Applicability	Broad	Restricted

H, isoniazid; R, rifampicin; Z, pyrazinamide; E, ethambutol. Adapted from reference [15].

**Table 3:** Eligibility Criteria for Short Therapy of Pediatric Tuberculosis (TB).

Criteria	Eligible	Not Eligible
Age	≥3 months	<3 months
Severity	Non-severe	Severe
Resistance	None	Any type of resistance
Previous TB	No	Recent TB

**Table 4:** Practical implementation considerations across levels of care.

What to Do	Who Does It	Where It Happens	Key Tools
Confirm eligibility for short-course therapy	Pediatric/TB clinician, district hospital team	Secondary/tertiary facility or district hospital	Chest radiography, microbiology where available, severity classification aid
Refer children and young adolescents with severe disease or diagnostic uncertainty	PHC provider	PHC to district/referral hospital	Referral criteria, referral form, phone or teleconsultation support
Back-refer stable non-severe cases for treatment continuation	District clinician or TB focal point	District hospital to PHC	Back-referral note, treatment card, follow-up plan
Deliver treatment and routine follow-up	PHC team, community health worker	PHC or community level	Standard treatment card, adherence checklist, caregiver counselling tools
Provide mentorship and case review	District/referral team	Hub-and-spoke network	Supportive supervision visits, remote case review, training package
Monitor quality and continuity of care	TB programme manager, district team	District or programme level	Audit tools, treatment outcome tracking, paper or electronic register

PHC, primary health care; TB, tuberculosis.

**Table 5:** Advantages vs. Limitations.

Domain	Advantages	Limitations
Adherence	Better	Still variable
Toxicity	Lower	Limited data
Cost	Lower	Implementation needed
Evidence	RCT support	Low certainty

RCT, randomized controlled trial.

## Data Availability

No new data were created or analyzed in this study. Data sharing is not applicable to this article.
